# Exploration of Phosphoproteins in *Acinetobacter baumannii*

**DOI:** 10.3390/pathogens14080732

**Published:** 2025-07-24

**Authors:** Lisa Brémard, Sébastien Massier, Emmanuelle Dé, Nicolas Nalpas, Julie Hardouin

**Affiliations:** 1 University of Rouen Normandy, INSA Rouen Normandie, CNRS, Polymers, Biopolymers, Surfaces Laboratory UMR 6270, 76000 Rouen, France; lisa.bremard1@univ-rouen.fr (L.B.); sebastien.massier@univ-rouen.fr (S.M.); emmanuelle.de@univ-rouen.fr (E.D.); 2 University of Rouen Normandy, INSERM US 51, CNRS UAR 2026, HeRacLeS-PISSARO, Normandie University, 76000 Rouen, France

**Keywords:** *Acinetobacter baumannii*, phosphorylation, proteomic, biofilm, virulence

## Abstract

*Acinetobacter baumannii* is a multidrug-resistant bacterium that has gained significant attention in recent years due to its involvement in a growing number of hospital-acquired infections. The World Health Organization has classified it as a critical priority pathogen, underscoring the urgent need for new therapeutic strategies. Post-translational modifications (PTMs), such as phosphorylation, play essential roles in various bacterial processes, including antibiotic resistance, virulence or biofilm formation. Although proteomics has increasingly enabled their characterization, the identification of phosphorylated peptides remains challenging, primarily due to the enrichment procedures. In this study, we focused on characterizing serine, threonine, and tyrosine phosphorylation in the *A. baumannii* ATCC 17978 strain. We optimized three parameters for phosphopeptide enrichment using titanium dioxide (TiO_2_) beads (number of enrichment fractions between the phosphopeptides and TiO_2_ beads, the quantity peptides and type of loading buffer) to determine the most effective conditions for maximizing phosphopeptide identification. Using this optimized protocol, we identified 384 unique phosphorylation sites across 241 proteins, including 260 novel phosphosites previously unreported in *A. baumannii*. Several of these phosphorylated proteins are involved in critical bacterial processes such as antimicrobial resistance, biofilm formation or pathogenicity. We discuss these proteins, focusing on the potential functional implications of their phosphorylation. Notably, we identified 34 phosphoproteins with phosphosites localized at functional sites, such as active sites, multimer interfaces, or domains important for structural integrity. Our findings significantly expand the current phosphoproteomic landscape of *A. baumannii* and support the hypothesis that PTMs, particularly phosphorylation, play a central regulatory role in its physiology and pathogenic potential.

## 1. Introduction

*Acinetobacter baumannii* is an opportunistic Gram-negative pathogen often implicated in infections in at-risk patients. It was recently observed that up to 80% of *A. baumannii* isolated strains were resistant to carbapenems in various areas, including Eastern Europe, Africa and South America [1]. Moreover, pandrug resistant clinical isolates are increasingly reported. Carbapenem-resistant *A. baumannii*, since 2017, has been classified by the World Health Organization (WHO) as among the “critical” pathogens for which the investigation of new therapeutic solutions is a priority [2]. *A. baumannii* is thriving and persisting in hospitals due to its genome plasticity (α parameter of Heap’s law = 0.71; [3]) allowing the acquisition of antibiotic resistance and its ability to form biofilms, enabling the colonization of biotic and abiotic surfaces [4].

Post-translational modifications (PTMs) of proteins are a mechanism used by all living organisms, including prokaryotes, to regulate all their biological functions. So far, over 500 different PTMs have been discovered [5] and can range from the addition of simple chemical groups to more complex molecules on a residue side chain of a protein. Modifications can have an impact on the three-dimensional structure by changing the residue charge or through steric hindrance, which then modulates protein activity, interactions (e.g., DNA–protein, protein–protein) or protein stability [6].

O-phosphorylation is a reversible and well-studied PTM occurring on serine (S), threonine (T) and tyrosine (Y) [7]. Its addition (+79.9663 Da) is catalyzed by kinases and its removal by phosphatases. Serine and threonine phosphorylation is largely catalyzed by Hanks-type kinases, which are also found in eukaryotes cells [8]. In contrast, tyrosine phosphorylation is mostly catalyzed by BY kinases (bacterial tyrosine kinases) [7]. In eukaryotes, it has been shown that phosphorylation plays an important role in cancer. For example, it has been demonstrated that the T286 mutation of cyclin D1 into a non-phosphorylable residue impacts the cyclin nuclear export in primary human cancer cells and thus allows neoplastic development [9]. So far, more than 70 small-molecule kinase inhibitors have been approved for cancer therapy [10].

For the same reason, the study of phosphorylation in bacteria is a prime area of research to renew therapeutic targets. Bacterial protein phosphorylation was first observed in 1978 by Wang and Koshland, who identified at least four phosphorylated proteins in *Salmonella typhimurium* [11]. Many previous studies have shown that bacterial phosphorylation is involved in all biological mechanisms [6]. In *Mycobacterium tuberculosis*, it has been demonstrated that the phosphorylation of the virulence factor CFP10 modulates the virulence and survival of bacteria by regulating the ESAT6 virulence mediator [12].

The first large-scale study of the *A. baumannii* phosphoproteome was carried out on the reference strain ATCC 17978 and the highly invasive multidrug-resistant strain Abh12O-A2 [13]. A higher number of phosphorylation sites was identified in Abh12O-A2 compared to ATCC 17978, which suggested that phosphorylation may contribute to the phenotypic differences between the two strains. In another comparative phosphoproteomic study between SK17 sensitive and resistant *A. baumannii* strains, Lai and colleagues [14] showed the impact of phosphorylation on drug-resistance mechanisms. The beta-lactamase AmpC, which is involved in ampicillin resistance, was found to be more phosphorylated in the sensitive strain than in the resistant one. The role of phosphorylation on S90 was established by direct mutagenesis, revealing a higher *β*-lactamase activity in the non-phosphorylated S90A mutant. A recent article demonstrated the impact of phosphorylation in *A. baumannii* resistance modulation with the phosphorylation of OXA 24/40 on its active site (S81), resulting in carbapenemase activity inhibition [15]. In 2021, the first large-scale phosphoproteome analysis of the extracellular proteins of *A. baumannii* was performed for the strain ATCC 17978 and the clinical isolate AB0057, in both biofilm and planktonic modes of growth [16]. Several extracellular proteins were observed to be phosphorylated, such as Hcp, a protein important in the type VI secretion system (T6SS). The authors showed that Hcp is secreted upon phosphorylation of S18.

Mass spectrometry (MS) is the method of choice to detect PTMs due to its high sensitivity, mass accuracy and resolution. However, detecting phosphorylation remains a challenge in bacteria due to inherent difference between bacteria and eukaryotes. As reported in the literature, up to 75% of eukaryotic proteins are phosphorylated in comparison to few percent in bacteria [17]. Since modified peptides are present at much lower stoichiometry compared to unmodified peptides [18], enrichment methods are required prior to MS analysis. Currently, the enrichment of phosphorylated peptides is based on ion metal or metal oxide affinity chromatography (IMAC or MOAC) [19], such as that using titanium dioxide (TiO_2_). However, this enrichment step needs to be optimized to improve phosphopeptide trapping. In fact, parameters such as the contact time between peptides and TiO_2_ [20], peptide amount [18,21] and loading buffer composition [18,21] have all been reported in the literature to significantly influence the number of identified phosphorylated peptides.

In this study, we investigated these phosphopeptide enrichment parameters to improve the detection of phosphopeptides. We defined the best parameters that enabled the recovery of the highest number of phosphopeptides in *A. baumannii* ATCC 17978. We then discussed the phosphorylated proteins and the hypothetical impact of their modified residues in the context of bacterial biology.

## 2. Materials and Methods

### 2.1. Strains and Growth Conditions

For this study, the *A. baumannii* strain ATCC 17978 (lacking the pAB3 plasmid, confirmed using PCR with previously described pAB3-specific primers) (Weber et al., 2015 [22]) was used. Strain grew overnight in Mueller-Hinton Broth II (MHB II, Difco) at 37 °C with shaking. Bacterial cultures were inoculated with approximately 1 × 10^7^ Colony Forming Units (CFU)/mL of overnight cultures. Cultures were grown in 50 mL of MHB II and incubated at 37 °C for 24 h with vigorous shaking (140 rpm).

### 2.2. Protein Extraction

Bacteria were harvested by centrifugation (8000× *g* for 15 min at 4 °C). The bacterial pellet was resuspended in 10 mL of 20 mM Tris-HCl buffer (pH 7.4) supplemented with a protease inhibitor cocktail (50 µL, HaltTM Protease and Phosphatase Inhibitor Single-Use Cocktail, ethylenediaminetetraacetic acid (EDTA)-Free (100×), Thermo Scientific, Waltham, MA, USA), and the histone deacetylase (HDAC) inhibitors nicotinamide (50 μL at 2 M, inhibitor of HDAC class III) and Trichostatin A (5 μL at 0.3 mM, inhibitor of HDAC classes I and II) (Sigma-Aldrich, Saint Louis, MO, USA) were added to each sample. The mixture was freeze–thawed for three cycles and then sonicated on ice six times, each for 1 min. The lysate was centrifuged at 9000× *g* for 30 min at 4 °C. An ultracentrifugation (Optima L-90K, Beckman Coulter, Brea, CA, USA) was performed, and the supernatant was collected (200,000× *g*, 45 min, 4 °C) to recover the cytoplasmic (soluble) proteins. Protein concentrations were evaluated by Bradford analysis (Bio-Rad, Hercules, CA, USA). Samples were stored in aliquots at −20 °C until further use.

### 2.3. Phosphopeptide Enrichment

Amount of 2 mg or 500 µg of proteins were dissolved and denatured in 6 M urea and 2 M thiourea, reduced with 5 mM (DL-dithiothreitol) DTT for 1 h at room temperature and carbamidomethylated with 15 mM iodoacetamide for 45 min at room temperature in the dark. Urea was diluted by addition of ammonium hydrogen carbonate (10 mM), and overnight digestion as completed at 37 °C with trypsin added at a 1:50 ratio. The peptide mixture was then acidified with TFA to pH < 3 and desalted (Sep-Pack column (3cc 50 mg). The peptide quantity was determined using the Pierce Quantitative peptide assays and standards (Thermo Fisher Scientific, Waltham, MA, USA). Phosphopeptide enrichment was performed using MOAC with TiO_2_ beads with a peptide/TiO_2_ ratio of 1/10 (Carlo Erba, Cornaredo, Italy), as previously described [23,24]. Briefly, the peptides are brought into contact with TiO_2_ for the specified time. After the contact time, the peptide–TiO_2_ solutions were centrifuged (3400× *g*, 2 min) and the supernatant was transferred to a new tube of TiO_2_. This operation is repeated according to the required number of fractions. In this context, 4 contact times between TiO_2_ and peptides were tested: 2 × 2 h, 4 × 1 h, 8 × 30 min and 16 × 15 min. In addition, 3 loading buffers were tested: 2,3-dihydroxybenzoic acid (DHB, 5 mg/mL in 80% ACN/0.1% trifluoroacetic acid (TFA)); glutamic acid (GA, saturated solution in 65% ACN/2% TFA) and TFA (80% ACN/6% TFA). After enrichment, phosphopeptides were dried out and conserved at −20 °C before use.

### 2.4. Tandem Mass Spectrometry

The enriched phosphopeptides were then analyzed using an Orbitrap Eclipse Tribrid mass spectrometer coupled to an EASY-nLC 1 200 (Thermo Scientific). Phosphopeptides were solubilized in FA 1% (7 µL) and 6 µL was injected onto an enrichment column (C18 PepMap100, Thermo Scientific). The separation was performed with an analytical column needle (NTCC-360/100-5-153, NikkyoTechnos, Tokyo, Japan). The mobile phase consisted of H_2_O/0.1% formic acid (FA) (buffer A) and CH_3_CN/FA 0.1% (buffer B). Phosphopeptides were eluted at a flow rate of 300 nL/min using a three-step linear gradient from 2 to 45% B over 121 min, from 45 to 100% B in 1 min and 9 min at 100% B. The mass spectrometer was operated in positive ionization mode with capillary voltage and source temperature set at 1.9 kV and 275 °C, respectively. The fragmentation mode was HCD (higher-energy collisional dissociation) with a collision energy of 28 eV. The first scan (MS spectra) was recorded in the Orbitrap analyzer (R = 60,000) with the mass range *m*/*z* 400–1800. Then, the 20 most intense ions were selected for MS^2^ experiments (R = 15,000). Singly charged species were excluded for MS^2^ experiments. Dynamic exclusion of already fragmented precursor ions was applied for 30 s, with a repeat count of 1, a repeat duration of 30 s, and an exclusion mass width of ±5 ppm. All measurements in the Orbitrap analyzer were performed with on-the-fly internal recalibration (lock mass) at *m*/*z* 445.12003 (polydimethylcyclosiloxane).

### 2.5. Database Searches

Raw data files were processed using Proteome Discoverer 1.4 software (Thermo Scientific). Peak lists were searched using MASCOT v. 2.6.0 search software (Matrix Science, London, UK) against the protein database *A. baumannii* ATCC 17978, containing 4097 protein sequences (Reference Sequences: NC_009085.1, NC_009083.1 and NC_009084.1; downloaded from http://www.genoscope.cns.fr, accessed on 12 September 2022). The search parameters were adapted to two missed trypsin cleavage sites allowed; variable modifications: carbamidomethylation of cysteine, oxidation of methionine and phosphorylation of serine, threonine and tyrosine. The parent ion and daughter ion tolerances were 5 ppm and 0.02 Da, respectively. The false discovery rate (FDR) threshold for identification was set at 1% (for proteins and peptides). Peptides were considered identified with a peptide ion score higher than 15, a peptide rank of 1 and both a q-value and an expectation value below 0.05. To avoid biased automatic annotation, all phosphopeptide spectra were manually inspected. The localization probabilities obtained from MASCOT and phosphoRS (Thermo Scientific) were used to unambiguously localize the phosphosites when possible.

### 2.6. Retrieval of Protein Functions

For each protein sequence, the reciprocal best hit method was used to obtain the corresponding unique accession identifier in different web resources, namely GenBank (i.e., A1S_XXXX), UniProt (i.e., 6 or 10 alphanumeric characters), VFDB (i.e., VFCXXXX), and CARD (i.e., ARO:XXXX). Notably, this allowed the retrieval of protein cellular localization (http://www.genoscope.cns.fr), Gene Ontology functional categories (UniProt), canonical pathways (KEGG) and virulence (VFDB) and resistance (CARD) information.

Interproscan v. 5.55-88.0 software [25] was used for protein sequence analysis to identify functional domains and conserved sites. eggNOG-mapper v. 2.1.8 software [26] was used on the protein entries from *A. baumannii* to retrieve functional annotation (COG categories) based on orthology. The protein three-dimensional structures were also searched with the online software Alphafold (https://alphafold.ebi.ac.uk/) [27] or RCSB Protein Data Bank (https://www.rcsb.org) [28]. When the structure was not referenced, protein alignments were performed to check for protein/residue conservation with NCBI BLAST (https://blast.ncbi.nlm.nih.gov/Blast.cgi (accessed on 21 July 2025)) [29].

## 3. Results

### 3.1. Optimization of the A. baumannii Phosphopeptide Enrichment Protocol

**Figure 1:**
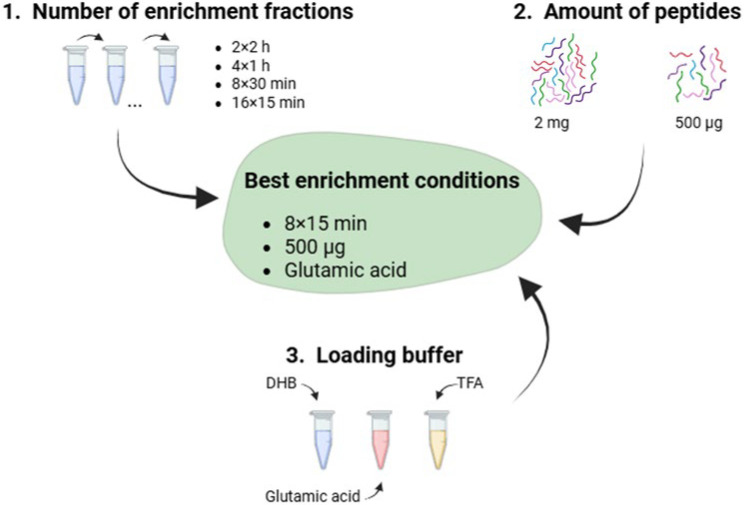
Illustration of the different parameters tested for phosphopeptide enrichment. The best enrichment condition is highlighted in green.

We tested different parameters for phosphopeptide enrichment in *A. baumannii*, namely the number of enrichment fractions between the phosphopeptides and TiO_2_ beads, quantity of peptides and type of loading buffer (Figure 1).

#### 3.1.1. Impact of the Number of Enrichment Fractions

**Figure 2:**
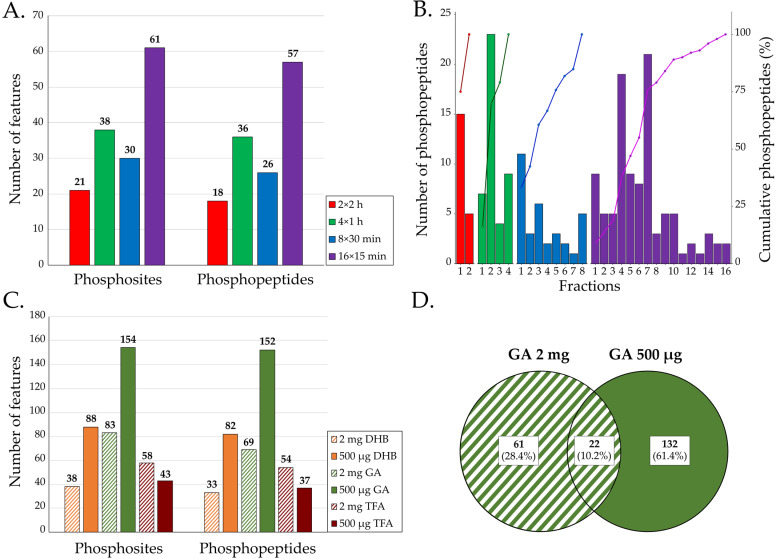
(**A**) The number of phosphosites and phosphopeptides detected as a function of the number of fractions according to the 2 × 2 h, 4 × 1 h, 8 × 30 min and 16 × 15 min methods (*n* = 1). (**B**) Number of phosphopeptides in each fraction and cumulative percentages of phosphopeptides according to the methods (*n* = 1). (**C**) Number of phosphopeptides and phosphosites based on both the peptide amount and the loading buffer (*n* = 2). (**D**) Number of phosphosites identified with GA according to the peptide quantity (*n* = 2).

We compared the impact of the number of enrichment fractions for a total contact time of 4 h between TiO_2_ beads and phosphopeptides with a DHB loading buffer: 2 × 2 h, 4 × 1 h, 8 × 30 min and 16 × 15 min. The retained phosphopeptides were analyzed by nanoLC-MS/MS. Following identification, all phosphopeptides were manually verified to confirm both the peptide sequences and phosphosite locations. Mass spectra that did not contain enough information (for sequence or localization) were removed (Table S1). The results, presented in Figure 2A, show that the 16 × 15 min method resulted in the highest number of identified phosphosites (61 sites) and phosphopeptides (57 peptides), followed by the 4 × 1 h method (36 peptides). Only six phosphosites were common to the four experiments, showing, as expected, the variability of phosphopeptide enrichment (Figure S1A) [18,30,31,32].

Next, we looked at the number of phosphopeptides recovered from each fraction. In the four experiments, the first fractions were those with a higher number of phosphopeptides identified (Figure 2B). A total of 79% of the phosphopeptides were harvested after eight enrichment fractions for the 16 × 15 min condition, 76% after five fractions for the 8 × 30 min condition, 79% after three fractions for the 4 × 1 h condition and 75% in one fraction for the 2 × 2 h condition. For all methods, the percentage of phosphopeptides/peptides ratios were below 0.3% (Figure S1B), showing that many non-phosphorylated peptides were recovered during phosphopeptide enrichment. The 16 × 15 min method had the highest ratio (0.27%), followed by the 4 × 1 h method (0.21%). The two other methods had ratios of around 0.13%. To know whether increasing the number of fractions could improve the number of phosphopeptides detected, we compared two methods (4 × 1 h and 6 × 1 h) using the 16 × 15 min as reference. With the 4 × 1 h method, we identified 63% of modified peptides compared with the 16 × 15 min condition, and 79% of modified peptides were recovered in the 6 × 1 h condition (Figure S1C). Increasing the number of fractions seems to have a benefit on the number of phosphopeptides detected.

These optimizations show that eight fractions of 15 min contact time yielded the highest number of phosphopeptides.

#### 3.1.2. Impact of Peptide Amount and Loading Buffer on Phosphopeptide Recovery

The impacts of peptide amount and loading buffer were assessed on the basis of eight fractions and a 15 min contact time. In this context, we compared 500 µg and 2 mg peptide mixtures and three different loading buffers: 2,5 dihydroxybenzoic acid (DHB), glutamic acid (GA) and trifluoroacetic acid (TFA). These agents minimize the interaction of contaminant peptides (e.g., acidic peptides) with TiO_2_ while preserving the binding of phosphopeptides.

More phosphosites were obtained with 500 µg of peptides using DHB or GA loading buffer (Figure 2C). With TFA, a higher number of phosphosites was found with 2 mg of peptides. Twenty-two phosphopeptides were shared between the 2 mg and 500 µg peptides inputs when using the GA loading buffer (Figure 2D). In addition, both the TFA 500 µg 8 × 15 min and GA 500 µg 8 × 15 min displayed an overlap of 54% between the two replicates (Table S2). Therefore, the use of GA with 500 µg of peptides appeared to be the best choice and enabled the identification of 154 phosphosites corresponding to 152 phosphopeptides.

#### 3.1.3. Comparison with Previous *A. baumannii* Phosphoproteomes

**Figure 3:**
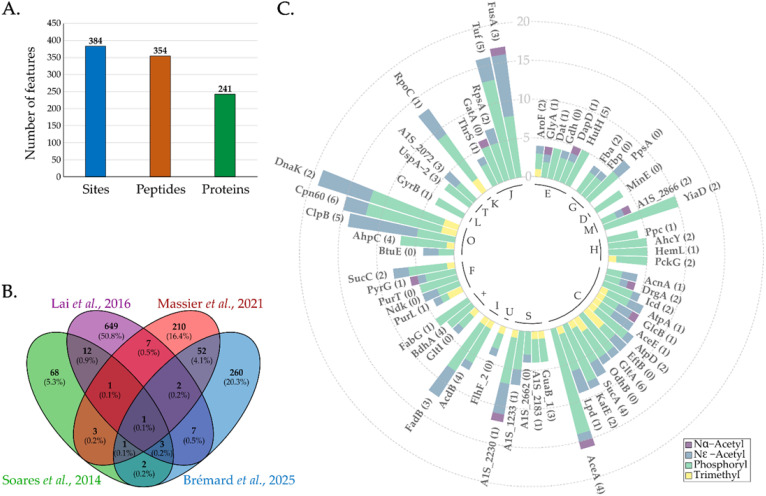
(**A**) Number of phosphosites, phosphopeptides and phosphoproteins identified overall in this study. (**B**) Comparison of the phosphosites identified in our study and the previous *A. baumannii* phosphoproteomes [13,14,16]. (**C**) Number of modified sites per PTM type for the most modified proteins identified in our data and in other studies. The number in parentheses indicates the number of novel phosphosites found in our study. The inner circle represents the following abbreviated functional categories: C: Energy production and conversion; D: Cell cycle control, cell division, chromosome partitioning; E: Amino acid transport and metabolism; F: Nucleotide transport and metabolism; G: Carbohydrate transport and metabolism; H: Coenzyme transport and metabolism; I: Lipid transport and metabolism; J: Translation, ribosomal structure and biogenesis; K: Transcription; L: Replication, recombination and repair; M: Cell wall/membrane/envelope biogenesis; O: Post-translational modification, protein turnover, chaperones; S: Function unknown; T: Signal transduction mechanisms; U: Intracellular trafficking, secretion and vesicular transport; +: Multiple functions.

Overall, we successfully identified a total of 384 phosphosites distributed on 241 proteins (Figure 3A, Table S3). We compared our dataset with the previously published phosphoproteomes of different *A. baumannii* strains: the reference strain ATCC 17978 [13,16], the highly invasive multidrug-resistant strain Abh12O-A2 [13], the SK17 sensitive and resistant strains [14] and the virulent multidrug-resistant AB0057 [16]. The identifications obtained by Soares et al. [13], Lai et al. [14] and Massier et al. [16] accounted for 91, 682 and 277 phosphosites, respectively. In our study, we only considered the 328 non-ambiguous phosphosites (Figure 3B). Only one phosphosite (PpsA on T416, Table S2) was found in all these studies. The limited overlap was expected due the different strains, culture conditions, analytical workflows and data processing methods used (Table S4) [18,30,31,32]. Moreover, six proteins are found to be common across the four large-scale studies (Figure S2A, Table S3). These proteins are implicated in translation (RpsA, A1S_1572), energy (PpsA, A1S_ 2164) and lipid metabolism (FabB), alginate production (AlgC; A1S_0887) and an unknown function (A1S_2230). As mentioned above, PpsA is found to be modified, as previously observed [13,14,16] (such as AlgC), while new sites have been identified for the other four proteins.

Thus, our study provides 260 novel phosphosites and 115 proteins never previously described as phosphorylated in *A. baumannii*. The percentage of S, T and Y phosphorylation was 43%, 40% and 17%, respectively (Figure S2B). This was comparable to the studies from Massier et al. [16] and Lai et al. [14]. However, a higher percentage of phosphoserine was found in the study by Soares et al. [13].

Nearly 70% of proteins were phosphorylated at only one site, whereas elongation factor Tu (A1S_0279) was identified as having 12 phosphosites (Figure S2C). We then retrieved additional datasets focusing on other modifications (Figure 3C, Table S2). Within our dataset, 146 proteins were previously reported as modified, i.e., phosphorylation [13,14,16], Nα-acetylation [33], Nε-acetylation [33] and lysine trimethylation [34]. Among these proteins, DnaK, FusA, AceA, EF-Tu and Cpn60 were among the top five most modified proteins. For them, we identified two, three, four, five and six new phosphosites, respectively (Figure 3C, Table S2). In addition, GlcB and A1S_2230 carried all four types of PTM.

### 3.2. Biological Identification of Phosphosites in A. baumannii

In the *A. baumannii* ATCC 17978 strain, 24 protein kinases have been reported (Genoscope, UniProt, NCBI). The vast majority (83%) are histidine kinases belonging to different two-component systems and with roles in biofilm formation (BfmS), efflux pump expression (BaeS), type IV pilus regulation (PilS), etc [35,36,37]. The three S/T/Y kinases reported in *A. baumannii* are much less characterized. The protein Wzc is a BY-kinase (essential protein) that is implicated in capsular polysaccharide export and exopolysaccharide synthesis [38]. The toxin HipA is a serine–threonine protein kinase that is known in several bacteria to induce persistence and mediate drug tolerance [39,40]. The A1S_2240 protein is a putative SPS1-like serine–threonine protein kinase with an unknown role in *A. baumannii*. While our data do not allow direct attribution of specific phosphorylation events to individual kinases, it is possible that these kinases are responsible for the addition of some phosphorylation sites identified in our study. For example, HipA has been shown in *E. coli* to phosphorylate proteins involved in translation, transcription and replication [40]. Further experiments, including kinase–substrate mapping and phosphoproteomic profiling in kinase-deficient mutants, will be essential to definitively link these phosphorylation events to their respective kinases.

After bioinformatics analysis and annotations, the 241 modified proteins belonged to 18 different functions (Figure S3A). As expected, the three most represented functions, which accounted for 36% of the proteins, were “unknown function”, “Energy production and conversion” and “Amino acid transport and metabolism”. Interestingly, 34 proteins displayed a phosphorylated site in a functional site, including 18 at multimer interfaces, 4 that were involved in structural stability and 23 in substrate-binding sites (Table S2, Figure S3B). Besides AlgC and GalE, which have already been described as phosphorylated at their active sites [14,16], we identified two novel phosphosites (S222 and S224) on EF-Tu (A1S_0279) that were involved in transfer ribonucleic acid (tRNA) binding [41] and one phosphosite on EngB (also known as YscX, A1S_3087) at S91 that involved GTP/Mg^2+^ binding [42]. Of the 241 proteins, many do not have functional sites described, which does not rule out the possibility that the residues modified in our study may be of interest. Further experiments will be needed to determine whether they are involved in functional sites (i.e., 3D structure, binding to another molecule). We then discussed selected phosphorylated proteins in the context of their biological function and the potential impact of their modified residues.

#### 3.2.1. Phosphorylated Proteins Involved in Protein Synthesis and Cell Division

**Figure 4:**
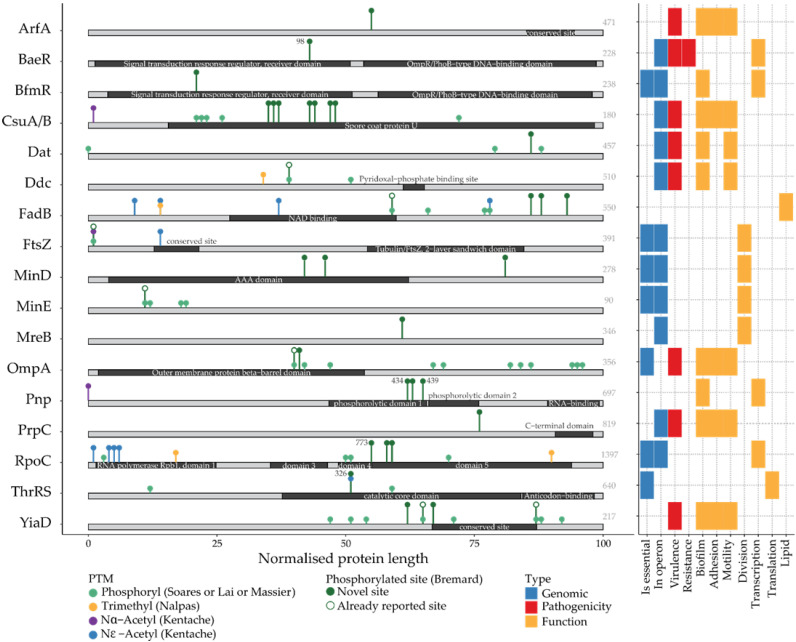
General overview of selected S/T/Y phosphorylated proteins. (left) The phosphorylated proteins discussed in this study are displayed as linear sequences. The modified sites are marked along the protein sequences based on our and previous studies [13,14,16,33,34]. The PTMs are represented as colored circles according to the PTM type (i.e., phosphorylation, trimethylation, acetylation). Modified residues within known functional residues are indicated with their exact sequence position. The functional domains are displayed along each protein sequence. (right) The heatmap represents the functional annotation for the same proteins. For these proteins, the presence of colored boxes indicates membership within specific genomic, pathogenic or functional categories.

In all living organisms, genomes are expressed in the cell through several mechanisms, allowing the decoding of genetic information into functional molecules. In bacteria, these mechanisms are temporally or physically coupled [43]. The proteins involved in these mechanisms, because of their importance, have been used as prime antibiotic targets (e.g., actinomycin, tetracycline). In our study, we identified several proteins involved in transcription, amino acid transfer, translation, protein maturation and degradation (Table S3). The phosphorylations found on three proteins are worth discussing in more detail due to their localization within functional sites (Figure 4 and Figure S3B). The ribonucleic acid (RNA) polymerase ꞵ′ subunit (RpoC, A1S_0288) was found to be modified in the ꞵ-ꞵ′ subunit interface at T773 [44]. This modification may regulate the interaction between the ꞵ and ꞵ′ subunits. In other studies, this protein was also found to be highly modified (phosphorylated, acetylated and trimethylated) [13,14,33,34]. The threonine-tRNA ligase (ThrRS, A1S_0592) catalyzes the attachment of threonine to tRNA via ATP hydrolysis and is the target of several bioactive molecules [45]. The phosphorylation is located on a residue (Y326) that is important for the homodimer interaction [46]. This residue has also been observed to be phosphorylated in *Shigella flexneri* [47]; this suggests it is important for the interaction. The PNPase, polynucleotide phosphorylase protein Pnp (A1S_0361), is implicated in messenger ribonucleic acid (mRNA) degradation. Interestingly, in a Δ*pnp E. coli* mutant, an overexpression of poly-*N-*acetylglucosamine (PNAG) was observed, resulting in cellular aggregation and biofilm production [48]. Pnp carried three phosphorylations, notably on the phosphate binding site (S439) and on a homotrimer interface site (S434) [49]. The modification of this protein on S439 may be linked to its normal activity, whereas S434 could regulate subunit organization.

Bacterial division is characterized by the differentiation of an internal structure in the mother cell, creating a partition that separates the two future daughter cells. The central point of this processus is the septal ring formed by a superposition of FtsZ proteins (A1S_3331). This protein was phosphorylated on the S3 residue. And while the phosphorylation is located within the N-terminal variable region, it has been previously observed in ATCC 17978 and Abh12O-A2 [13]. The positioning of the septal ring in the midcell requires three proteins encoded by the *minCDE* operon [50]. The MinD ATPase (A1S_0880) allows the recruitment of MinC to the polar region of the cell. In turn, MinC inhibits Z-ring formation in these regions. MinD was phosphorylated on three residues, of which two, S128 and S224, are located within one residue of the P-loop NTPase active sites (127 and 225) [51]. The presence of these phosphorylations could mask the active sites and alter the ATPase activity. In parallel, MinE (A1S_0879) enables the disassembly and pole-to-pole oscillation of MinCD polymers, thus enabling formation of the Z-ring. The phosphorylation observed on MinE (S10) has already been reported in *A. baumannii* [13,14] and in *E. coli* K12 [31]; its conservation suggests a potential role for the function of MinE. MreB (A1S_2781) is an homologous protein to actin that is implicated in bacterial cell shape and required for bacterial elongation [52]. It was phosphorylated in our data on T212 and is conserved in *E. coli* K12 [31]; its close proximity to the nucleotide binding site (215) could regulate the binding.

#### 3.2.2. Phosphoproteins Implicated in Fatty Acid Metabolism

**Figure 5:**
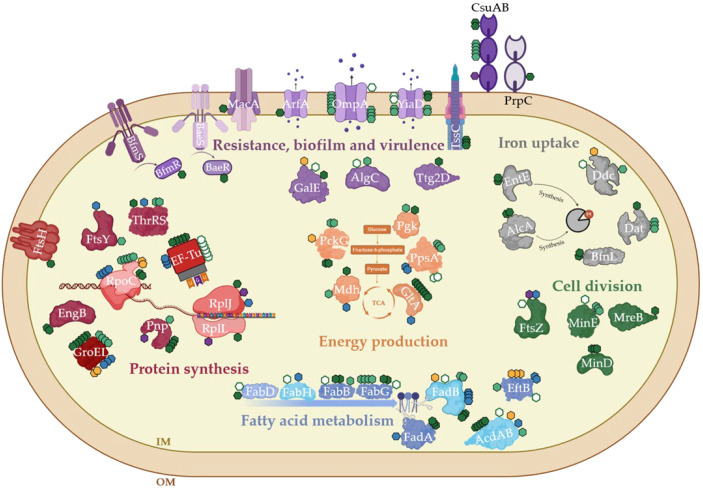
Illustration of all mechanisms involving phosphorylated proteins identified in our data. The PTMs are represented as colored hexagons according to the PTM type based on our own and previous studies [13,14,16,33,34]. Novel phosphorylation in dark green, already identified phosphorylation in white with green edge, phosphorylation from other studies in green, trimethylation in yellow, Nα-acetylation in purple and Nε-acetylation in blue.

Lipids are the major component of bacterial membranes. Fatty acids are important in different pathways in bacteria (resistance, virulence, biofilm, motility). In our study, we identified several proteins involved in fatty acid biosynthesis (AccC, FabD, FabH, FabB, FabG and AcdA/B) and degradation (AtoB, FadB and FadA) (Table S3). Some of the phosphorylated sites detected here were reported in previous phosphoproteomic studies [13,14,16]. However, in our study, we highlighted new phosphosites on these proteins. Moreover, we observed that many of the proteins involved in fatty acid biosynthesis or degradation could carry many other PTMs. FadB was previously described as phosphorylated at four sites: D429 and Y424 in the strain SK-17 sensitive [14], S363 in ATCC 17978 and Abh12O-A2 [13] and T322 in ATCC 17978 [16]. Here, in addition to T322, we have identified three new phosphorylated residues, T474, T485 and T513, increasing our knowledge of PTMs of this protein (Table S2). The two last residues were phosphorylated on the same peptide, meaning that the two modifications are present at the same time. This protein was previously observed to be acetylated on K52, K77, K203 and K430 [33] and trimethylated on K77 [34] (Figure 4 and Figure 5). Our data provide new the phosphosites of proteins involved in fatty acid metabolism. This suggests that phosphorylations, and PTMs in general, can play a role in the modulation of this pathway in bacteria (which could affect the cell membrane [53], energetic metabolism [54], stress adaptation [55] or virulence [56]).

#### 3.2.3. Phosphorylation of Antibiotic Resistance, Biofilm Formation and Virulence Determinants

Of the various antibiotic resistance mechanisms possessed by *A. baumannii* [57], two seem to be particularly targeted by phosphorylation: antibiotic enzymatic modification and modifications of envelope permeability. Indeed, several phosphorylations on *β*-lactamases were already identified and shown to modulate the activity of the AmpC or Oxa24/40 enzymes [14,15,16].

In this study, we were able to identify phosphorylation on Y98 carried by the cytoplasmic regulator BaeR (A1S_2883), a protein belonging to the BaeR/S two-component system (TCS). By positively regulating the *adeAB* genes of the AdeABC efflux pump, BaeR/S is involved in tigecycline [58]. Interestingly, it was shown recently that the mutation S104N in BaeR is involved in cefiderocol resistance, probably via the overexpression of an MFS (Major Facilitator Superfamily) transporter [59]. Based on protein alignment and the *E. coli* BaeR structure [60], we are able to localize the residues S104 and Y98 in the α4-*β*5 swap domain of N-terminal receiver domain, which is involved in the BaeR dimerization required for its activity, suggesting the importance of these phosphorylations.

This study also identified several phosphorylations on three outer membrane proteins, OmpA (A1S_2840) and the OmpA-like proteins YiaD (A1S_0884) and ArfA (A1S_1193). They play important roles as adhesins in virulence and biofilm formation, and are also involved in *A. baumannii’s* antibiotic susceptibility—OmpA affects susceptibility to aminoglycosides, certain penicillins and fluoroquinolone, while YiaD is associated with susceptibility to meropenem [61,62,63]. The phosphorylations we identified on these proteins were mainly localized in their periplasmic C-terminal domains (Figure 4, Table S2), also called the OmpA-like domain. This domain anchored these outer membrane proteins to the peptidoglycan layer and contributed to maintaining the integrity of the bacterial envelope [64]. It was suggested that this domain may also interact with the inner membrane transporters of resistance-nodulation-division (RND) efflux pumps to participate in antibiotic extrusion [61,65]. Phosphorylations of this periplasmic domain may contribute to the interactions of OmpA and OmpA-like proteins with peptidoglycan or other membrane proteins, thus modulating their function.

As appendices mediating bacterial adhesion to biotic and abiotic surfaces, pili are major virulence traits of *A. baumannii* [66,67,68,69]. The type I pilus system Csu is formed of four subunits called CsuA/B, CsuA, CsuB and CsuE [70]. This pilus is an essential component of the *A. baumannii* mature biofilm and also promotes bacterial adhesion to epithelial cells [69]. CsuA/B (A1S_2218) is a multi-modified protein. Previous phosphoproteomic studies reported modified sites on the subunit’s N-terminal domain that were involved in the interaction with the CsuC chaperon and subunit polymerization [16]; this domain is also reported as being acetylated [33]. Here, two other sites were identified, localized on the B or B’ β-sheets of the structure near the disulfide bridge (ambiguous sites 64–66 and 77–80–85–87, Table S2) [70]. These phosphorylations may participate in the tight conformation of the subunit. In our data, we also found the fimbriae usher membrane protein PrpC (a.k.a. Caf1A and PapC, A1S_2089) to be phosphorylated at Y622 (Figure 4, Table S2). This pilus is shorter and thinner than the Csu pilus and implicated in the biotic adhesion of *A. baumannii* to human respiratory cells [71]. Interestingly, residue 622 is within the inner surface of the β-barrel and has been described as forming hydrophobic interactions with conserved residues of the UMD (usher middle domain) [72]. This phosphorylation may regulate the formation of this hydrophobic interaction with the UMD, which is essential for plugging or priming secretion through the usher channel [72].

Biofilm formation often rapidly follows bacterial adhesion and has been associated with *A. baumannii* virulence [73,74]. It is mainly regulated by the TCS BfmR/S (Biofilm Master Regulator, A1S_0748). This TCS is crucial in the early stages of *A. baumannii* biofilm or pellicle formation through its control of Csu pili expression [75]. The phosphorylation site permitting the activation and dimerization of BfmR is localized on D58 [75] in the response regulator receiver domain [76]. Interestingly, in our data, a phosphorylation site was identified on the same receiver domain but at S49 (Figure 4, Table S2). After modeling BfmR using the AlphaFold Protein Structure Database (AlphaFold A0A836M3P4), the modified residue S49 does not seem to be involved in the interaction with the key D58 phosphorylation site or DNA binding sites. Indeed, this modified residue, which is located on the *α*2 helix, is turned towards the outside of the protein and may contribute to its structural stability [75].

Finally, several proteins involved in virulence were phosphorylated in our study (e.g., TssC and GalE) (Table S3). Iron uptake and acquisition is also a known virulence pathway in *A. baumannii* when extracellular iron availability is low [77]. *A. baumannii* uses several systems to capture iron, such as the one involving the baumannoferrin gene cluster (*A1S_1647*-*A1S_1657*) [77]. Here, we found three proteins involved in baumannoferrin biosynthesis that were phosphorylated: Dat (A1S_2454; a.k.a. DABA-AT), Ddc (A1S_2453; a.k.a. DABA-DC) and BfnL (A1S_1657) [78,79]. Both the *dat* and *ddc* genes are required for *A. baumannii* motility and virulence in the *Galleria mellonella* infection model [78,79]. In our data, the Dat protein was found to be phosphorylated at S391, in the small domain of this PLP-dependent (pyridoxal-5-phosphate coenzyme) aminotransferase that functions as a dimer [80]. The Ddc protein was found to be modified on residue S201, localized in the major domain of the protein [81]. The phosphorylation may participate in the activity of these enzymes.

## 4. Conclusions

In this study, we tested the impact of several parameters on phosphopeptide recovery. The highest number of phosphopeptides was obtained in the condition using eight fractions of 15 min contact time between 500 µg of peptides and TiO_2_ and using glutamic acid in the loading buffer. Here, we identified 241 phosphoproteins increasing our knowledge of the phosphorylation landscape in *A. baumannii*. The phosphorylated proteins were involved in general bacterial metabolism and pathways of interest such as fatty acid-related pathways, biofilm formation, virulence and resistance. Moreover, we detected 46 phosphosites that are localized in functional sites, suggesting that phosphorylation can modulate protein activity, interaction or conformation. In the future, it will be essential to validate the functional impact of the phosphoresidues using residue-specific mutants (phosphorylation ablative and mimetic).

A comparison of the phosphorylated proteins identified here against previously published large-scale phosphoproteomic studies showed that over 50% of the proteins were identified in a single study. This highlights the need for more phosphoproteomic studies in different strains and growth conditions to capture the full diversity of phosphorylation events and better understand any differences observed. In comparison to other PTM studies, 98 proteins identified here as phosphorylated have previously been found as Nε-acetylated, Nα-acetylated or K-trimethylated. In the future, it would be interesting to investigate potential cross-talk between PTMs, especially in highly modified proteins.

In light of previous studies and our results, protein phosphorylation has far-reaching roles in bacteria, including in bacterial biofilm formation, resistance and virulence, making it a promising antimicrobial target.

## Data Availability

The MS proteomics data have been deposited in the ProteomeXchange Consortium via the PRIDE partner repository [82] with the dataset identifier PXD064268.

## Supplementary Materials

The following supporting information can be downloaded at: https://www.mdpi.com/article/10.3390/pathogens14080732/s1, Figure S1: Comparison of phosphopeptide recovery in terms of different numbers of enrichment fractions and contact times between TiO_2_ and the peptides; Figure S2: Comparison between large-scale phosphoproteome studies of *A. baumannii*; Figure S3: Function and important residues in phosphorylated proteins.; Table S1: Phosphorylated peptides identified in the intracellular medium of *A. baumannii* ATCC 17978; Table S2: Phosphorylated sites identified in the intracellular medium of *A. baumannii* ATCC 17978; Table S3: Phosphorylated proteins identified in the intracellular medium of *A. baumannii* ATCC 17978. Table S4: Comparison of the different experimental parameters used in the four phosphoproteomic studies of *A. baumannii*.

## Abbreviations

The following abbreviations are used in this manuscript:

AF	Formic acid
CFU	Colony forming units
DHB	2,3-Dihydroxybenzoic acid
DTT	DL-Dithiothreitol
EDTA	Ethylenediaminetetraacetic acid
FDR	False discovery rate
GA	Glutamic acid
HCD	Higher-energy collisional dissociation
HDAC	Histone deacetylase
IMAC	Immobilized metal affinity chromatography
MFS	Major facilitator superfamily
MHB II	Mueller-Hinton Broth II
mRNA	Messenger ribonucleic acid
MS	Mass spectrometry
OMAC	Metal oxide affinity chromatography
PCR	Polymerase chain reaction
PLP	Pyridoxal-5-phosphate
PTMs	Post-translational modifications
RNA	Ribonucleic acid
RND	Resistance-nodulation-division
S	Serine
T	Threonine
T6SS	Type VI secretion system
TCA	tricarboxylic acid cycle
TCS	Two-component system
TFA	Trifluoroacetic acid
TiO_2_	Titanium dioxide
tRNA	Transfer ribonucleic acid
UMD	Usher middle domain
WHO	World Health Organization
Y	Tyrosine
