# Supplementary material for: Exploration of Phosphoproteins in Acinetobacter baumannii
Source: Pathogens. 2025 Jul 24;14(8):732. doi: 10.3390/pathogens14080732 (PMC12388955; doi:10.3390/pathogens14080732)
Supplement: Supplementary file 1 [file pathogens-14-00732-s001.zip › Pathogens_article_supp_figures_notmarkedup.pdf]

Exploration of phosphoproteins in *Acinetobacter baumannii*

Lisa Brémard[1], Sébastien Massier[1], Emmanuelle Dé[1], Nicolas Nalpas[1,2,#,*], Julie Hardouin[1,2,#,*]

[1] University of Rouen Normandy, INSA Rouen Normandie, CNRS, Polymers, Biopolymers, Surfaces Laboratory UMR 6270, 76000 Rouen, France
[2] University of Rouen Normandy, INSERM US 51, CNRS UAR 2026, HeRacLeS-PISSARO, Normandie Université, 76000 Rouen, France
# The authors contributed equally to this article.
* Dr Julie Hardouin, Laboratoire Polymères, Biopolymères, Surfaces, UMR CNRS 6270, Université de Rouen, 76821 Mont-Saint-Aignan cedex, France. julie.hardouin@univ-rouen.fr
* Dr Nicolas Nalpas, Laboratoire Polymères, Biopolymères, Surfaces, UMR CNRS 6270, Université de Rouen, 76821 Mont-Saint-Aignan cedex, France. nicolas.nalpas@univ-rouen.fr

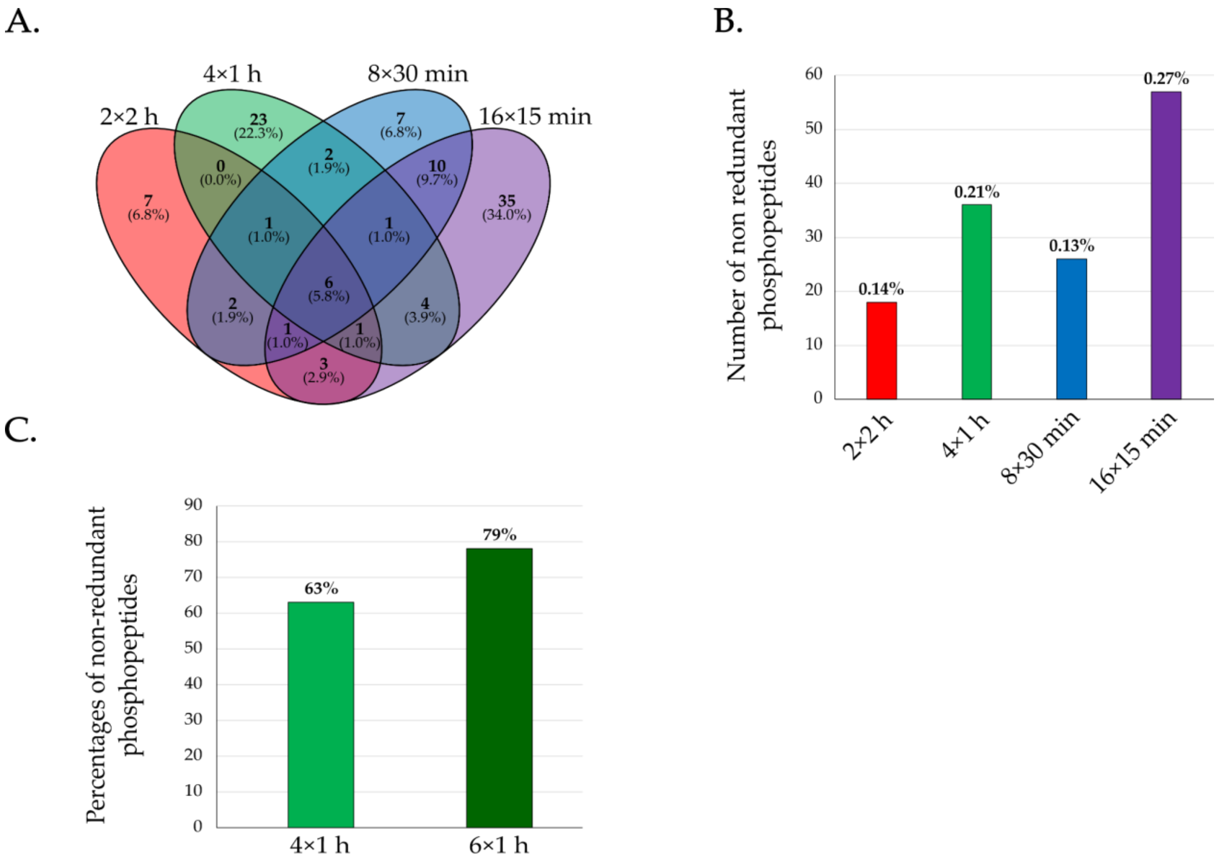

Figure S1. Comparison of phosphopeptides recovery in function of number of enrichment fractions and contact time between TiO_2 and peptides. (A) Comparison of phosphorylated sites identified in different strategies for contact time enrichment (n = 1). (B) For each contact time enrichment strategies, the number of non-redundant identified phosphopeptides and the ratio between phosphopeptides and total peptides is displayed (n = 1). (C) Percentage of non-redundant phosphorylated peptides in function of different number of fractions (100% represents the number of phosphorylated peptides in 16×15 min samples) (n = 1).

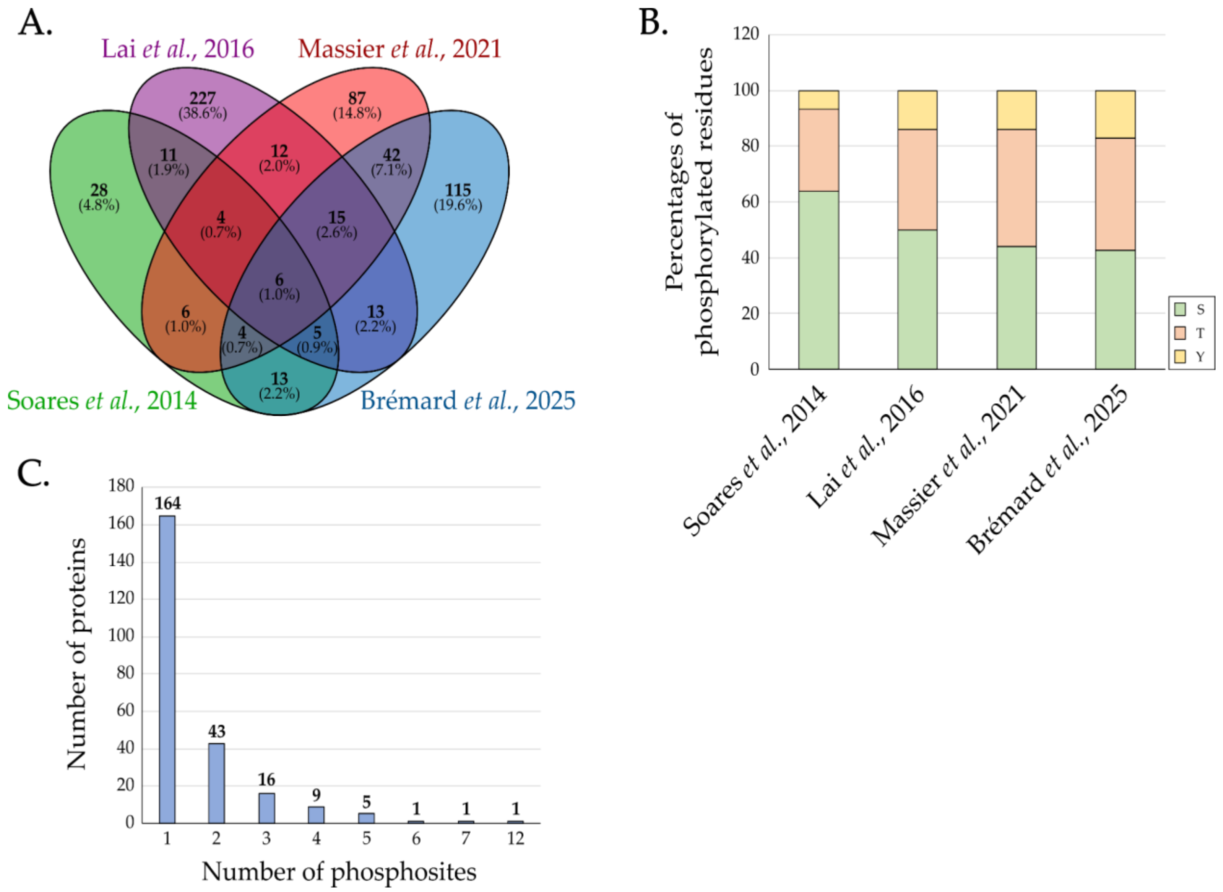

Figure S2. Comparison between large scale phosphoproteome studies of *A. baumannii*. (A) Number of phosphoproteins identified in our study and the previous *A. baumannii* phosphoproteomes [13,14,16]. (B) Percentages of phosphorylated S/T/Y residues in this study and the previous large-scale studies [13,14,16]. (C) Number of phosphosites per protein found in this study.

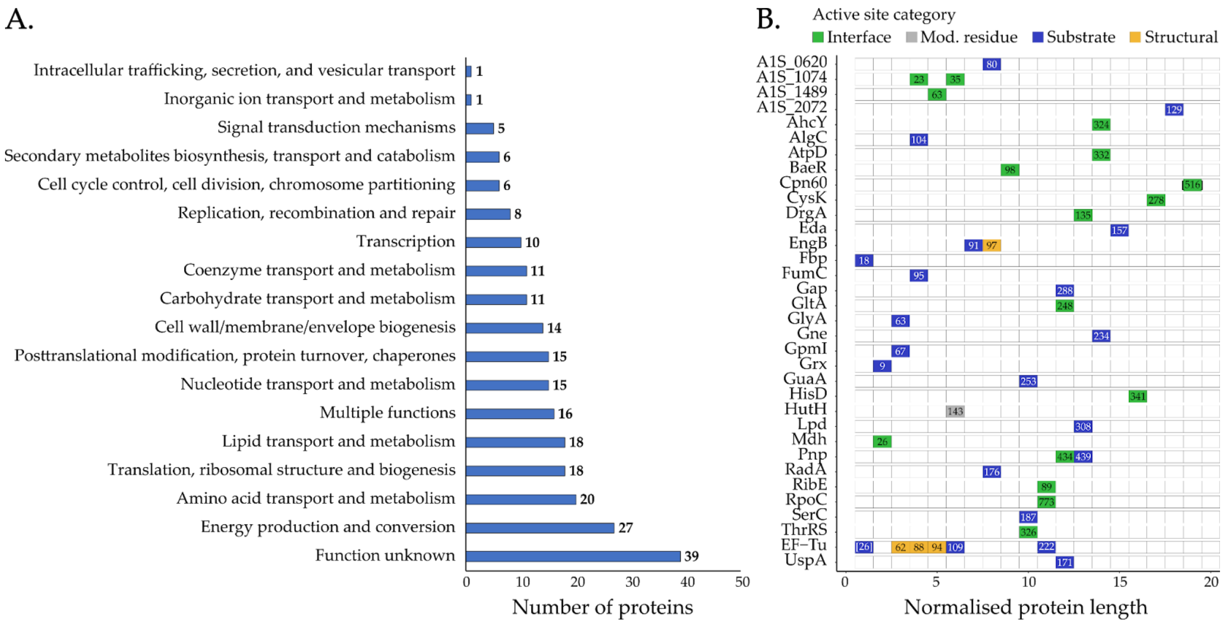

Figure S3. Phosphorylated proteins function and important residues. (A) The number of phosphorylated proteins in ATCC 17978 per functional categories. (B) Proteins found modified on functional sites. The functional sites are color coded based on their role in interface (in green), substrate binding (in blue), structural domains (in orange) and modified residues (in gray).
